# Comparative transcriptome profiling of the fertile and sterile flower buds of a dominant genic male sterile line in sesame (*Sesamum indicum* L.)

**DOI:** 10.1186/s12870-016-0934-x

**Published:** 2016-11-10

**Authors:** Hongyan Liu, Mingpu Tan, Haijuan Yu, Liang Li, Fang Zhou, Minmin Yang, Ting Zhou, Yingzhong Zhao

**Affiliations:** 1 Key Laboratory of Biology and Genetic Improvement of Oil Crops, Ministry of Agriculture, Oil Crops Research Institute of Chinese Academy of Agricultural Sciences, Wuhan, Hubei 430062 China; 2 College of Life Sciences, Nanjing Agricultural University, Nanjing, Jiangsu 210095 China

**Keywords:** Sesame, Dominant genic male sterile, Transcriptome, Differentially expressed genes

## Abstract

**Background:**

Sesame (*Sesamum indicum* L.) is a globally important oilseed crop with highly-valued oil. Strong hybrid vigor is frequently observed within this crop, which can be exploited by the means of genic male sterility (GMS). We have previously developed a dominant GMS (DGMS) line W1098A that has great potential for the breeding of F_1_ hybrids. Although it has been genetically and anatomically characterized, the underlying molecular mechanism for male sterility remains unclear and therefore limits the full utilization of such GMS line. In this study, RNA-seq based transcriptome profiling was carried out in two near-isogenic DGMS lines (W1098A and its fertile counterpart, W1098B) to identify differentially expressed genes (DEGs) related to male sterility.

**Results:**

A total of 1,502 significant DEGs were detected, among which 751 were up-regulated and 751 were down-regulated in sterile flower buds. A number of DEGs were implicated in both ethylene and JA synthesis & signaling pathway; the expression of which were either up- or down-regulated in the sterile buds, respectively. Moreover, the majority of NAC and WRKY transcription factors implicated from the DEGs were up-regulated in sterile buds. By querying the Plant Male Reproduction Database, 49 sesame homologous genes were obtained; several of these encode transcription factors (bHLH089, MYB99, and AMS) that showed reduced expression in sterile buds, thus implying the possible role in specifying or determining tapetal fate and development. The predicted effect of allelic variants on the function of their corresponding DEGs highlighted several Insertions/Deletions (InDels), which might be responsible for the phenotype of sterility/fertility in DGMS lines.

**Conclusion:**

The present comparative transcriptome study suggested that both hormone signaling pathway and transcription factors control the male sterility of DGMS in sesame. The results also revealed that several InDels located in DEGs prone to cause loss of function, which might contribute to male sterility. These findings provide valuable genomic resources for a deeper insight into the molecular mechanism underlying DGMS.

**Electronic supplementary material:**

The online version of this article (doi:10.1186/s12870-016-0934-x) contains supplementary material, which is available to authorized users.

## Background

Sesame (*Sesamum indicum* L.) is a globally important and ancient oilseed crop mainly consumed for high-quality oil [1, 2]. It has the highest oil content among the cultivated oil crops and is rich in natural antioxidants like sesamin and sesamol, which are known by their specific antihypertensive effects and anti-oxidative activity [3–5]. Although important, the seed yield of sesame is unstable and relatively low compared with rapeseed, peanut and soybean. Therefore, great efforts should be made to improve the seed yield of sesame.

Heterosis utilization is the most promising approach for yield improvement, since very strong hybrid vigor (>15 %) has been observed within this crop [6]. Heterosis can be effectively exploited either by cytoplasmic male sterility (CMS) or genic male sterility (GMS). So far, only recessive GMS has been successfully applied to the production of sesame F_1_ hybrids. However, this method might be constrained by certain drawbacks such as environmental sensitivity, incomplete sterility, and the timely removal of 50 % male-fertile plantlets from two-type lines for hybrid seeds production [7]. Recently, we have developed a novel dominant GMS line (DGMS) by crossing the wild species *S. mulayanum* L. (2n = 26) plants with the cultivated species *S. indicum* L. (2n = 26), which has great potential for the breeding of hybrid varieties. Cytological study showed that pollen abortion in the DGMS line (W1098A) began in pollen mother cells (PMC), continued throughout pollen development, and peaked at the late microspore stage. Moreover, the gene locus conditioning male sterile was delimited by two closely linked SSR markers SBM298 and GB50 [8]. However, the underlying molecular mechanism remains elusive.

The small diploid genome (~350 Mb) makes sesame an attractive species for genetic studies [9, 10]. Recently, the high-quality genome sequence of sesame was assembled, which contains ~27,148 predicted gene models, of which 91.7 % were anchored onto 16 pseudomolecules or linkage groups (LGs) [11]. Using forward and reverse genetic approaches, a growing number of genes have been identified that have vital roles in anther development. Consequently, the Plant Male Reproduction Database (PMRD, http://202.120.45.92/addb/), a comprehensive resource for genes and mutants related to plant male reproduction, has emerged [12].

Male sterility (MS) is associated with not only the lack of viable pollen, but also the failure of pollen release [13]. The importance of tapetal programmed cell death (PCD) for successful pollen formation has been highlighted by a number of MS mutants that fail to go through normal tapetal breakdown [13–15]. Archesporial cell number and tapetal cell fate is controlled by EXCESS MICROSPOROCYTES1 (EMS1), a leucine-rich repeat receptor like kinase, and a small secreted protein ligand, TAPETUM DETERMINANT1 (TPD1) [16]. Tapetal development is initiated by *DYSFUNCTIONAL TAPETUM1* (*DYT1*) [17] and *DEFECTIVE IN TAPETAL DEVELOPMENT AND FUNCTION1* (*TDF1*) [18], with tapetal maturation, pollen wall formation, and tapetal PCD involving *ABORTED MICROSPORES* (*AMS*) [19] and *MALE STERILITY1* (*MS1*) [20]. The final stage of dehiscence involves jasmonic acid (JA)-induced gene expression and transcription factors associated with endothecium secondary thickening [13].

To elucidate the mechanism of MS more comprehensively, the transcriptomes of many higher plants have been sequenced, including Arabidopsis [21], buckwheat [22], cotton [23–25], watermelon [26], soybean [27], *Brassica napus* [28–30] and *Brassica oleracea* [31]. In this study, fertile and sterile flower buds from DGMS line with a length of ~2.5 mm were sampled for RNA-seq, representing the first study of the sesame DGMS transcriptome. The aim of this study is to identify differentially expressed genes (DEGs) associated with MS, and explore the different bioprocesses involved and their putative functions. These results will be helpful to elucidate the molecular mechanism for DGMS, and assist the breeding of sesame hybrid variety.

## Results

### Transcriptome profiling of fertile and sterile buds

We have previously demonstrated that male sterility mainly occurred at PMC stage in DGMS line [8]. Therefore, we sampled fertile and sterile buds at this stage, and prepared respective cDNA libraries. After sequencing with Illumina HiSeq 2000 platform, we obtained a total of 53,126,890 and 55,491,408 high quality pair-end reads from fertile and sterile flower buds, respectively, which were then cleaned and mapped to the sesame reference genome sequence containing 27,148 gene models [11]. In total, 83.54 % of the reads from fertile buds and 84.86 % from sterile buds were mapped to the reference genome, and the majority of which were uniquely mapped (Table 1). By sequences alignment, we found that a total of 22,373 and 22,788 genes were hit by the unique reads from fertile and sterile buds, respectively, which accounted for >82 % of the known gene models. The average length of genes in fertile buds was 1305 bp and it was 1297 bp for sterile buds. Most of these genes (74 % in sterile buds and 71 % sterile buds) showed very high level of gene coverage (90–100 %).

**Table 1:** Summary of mapping transcriptome reads to reference sequence of sesame

	Fertile Buds		Sterile Buds	
	Quantity	Percentage %	Quantity	Percentage %
Total Reads	53,126,890	100.00	55,491,408	100.00
Total Mapped Reads	44,382,564	83.54	47091778	84.86
Unique Match	43,380,846	81.66	46141578	83.15
Perfect Match	36,189,950	68.12	37126510	66.90
≦5 bp Mismatch	8,192,614	15.42	9965268	17.96

To gauge the relative level of gene expression in different tissues, we calculated the RPKM (Reads per Kilobase of exon model per Million mapped reads) value based on the uniquely mapped reads. The RPKM value for those genes detected in fertile buds ranged from 0.012 to 16683.020, with a mean of 40.974. Similarly, the minimum, maximum and average RPKM was 0.008, 33521.52 and 40.302 for genes in sterile buds. Thus, all the above genes were regarded to be expressed in either the fertile buds or the sterile buds, as indicated by a RPKM threshold ≥0.001. Unsurprisingly, most of these expressed genes (>95 %) were common between tissues; however, we also observed a small number of uniquely expressed genes (539 in fertile buds and 954 in sterile buds).

### Functional characterization of DEGs

**Fig. 1:**
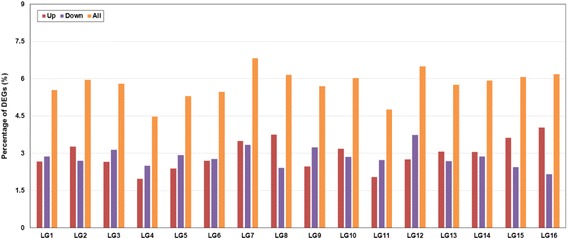
Percentage of differentially expressed genes in each linkage group. Up/Down: up-/ down- regulated DEGs in sterile buds; All: all of the DEGs; LG: linkage group

Using the criteria of at least two fold changes and false discovery rate (FDR)<0.001, we obtained 1,502 significant DEGs by comparing the genes expression levels between fertile and sterile buds, of which 751 were up-regulated and 751 down-regulated in sterile buds (Additional file 1: Table S2). Distribution of all DEGs across the sesame genome was then analyzed by anchoring gene sequences to the previously released 16 pseudomolecules (or LGs) that harbored 85.3 % of the sesame genome assembly [11]. By integrating the genome information available in public domain, we could assign the DEGs onto each LG. The results showed that LG4 had the least numbers of DEGs (4.47 %), following by LG11 with 4.76 %. In contrast, LG7 had the largest percentage of DEGs (6.83 %). Moreover, the percentage of up-regulated genes was nearly 2 folds that of down-regulated genes in LG16, LG8 and LG15. Also, LG2, LG10 and LG13 had higher percentage of up-regulated genes than down-regulated genes, while LG3, LG4, LG5, LG9, LG11 and LG12 showed an opposite trend. In addition, there were nearly equal numbers of up- and down- regulated genes in the rest of the four LGs (Fig. 1).

**Fig. 2:**
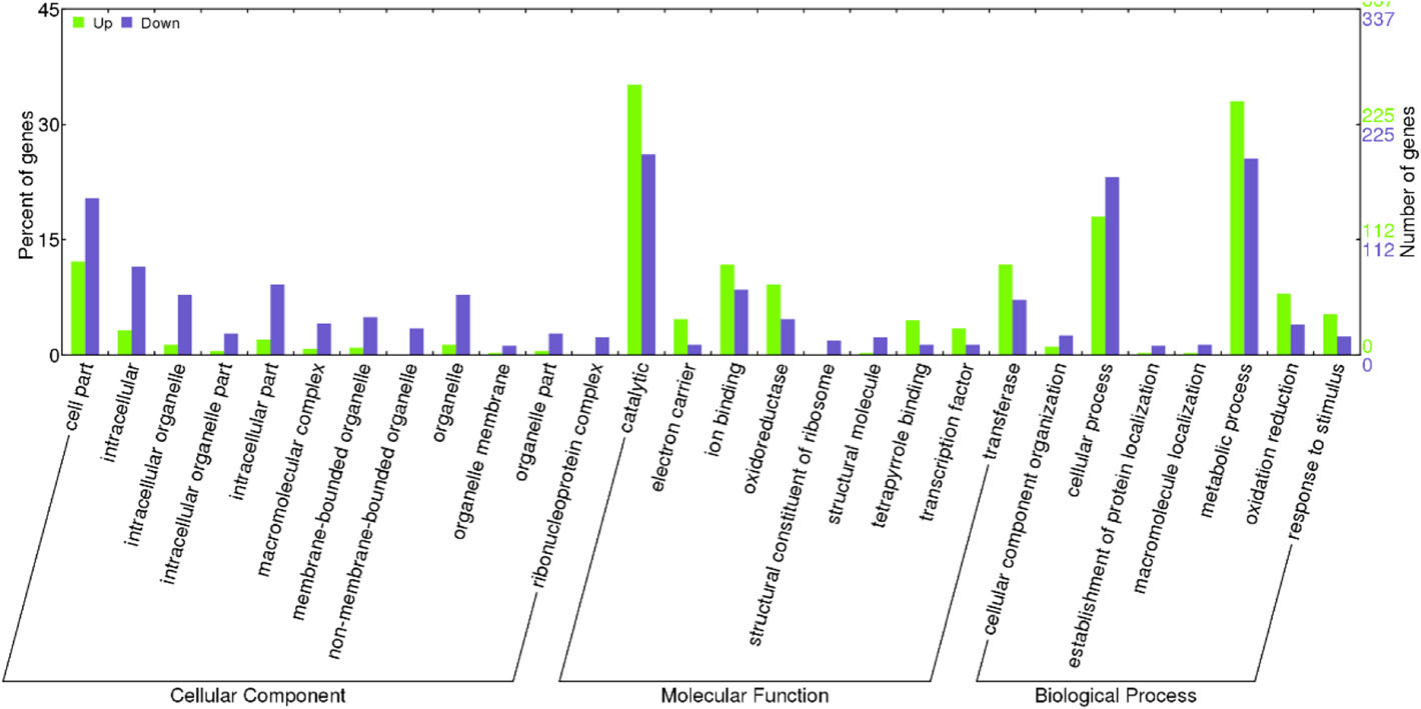
Classification of enriched GO terms of up- and down- regulated genes in sterile buds. The x-axis indicates the differentially expressed genes (DEGs) enriched sub-categories in three main categories: biological process, molecular function and cellular component by GO analysis, and the left y-axis indicates the percentage of DEGs of a sub-category in the main category and the right y-axis indicates the number of DEGs in a sub-category

The putative function of each DEG was then characterized with both GO (Gene Ontology) and KEGG (Kyoto Encyclopedia of Genes and Genomes) databases. Due to the large numbers and the complex branch structure of GO categories, only the three most abundant functional groups, namely ‘Cellular Component’, ‘Molecular Function’ and ‘Biological Process’ were presented, as an example (Fig. 2). In the sub-category of ‘Cellular Component’, the largest numbers of genes were found to be associated with ‘cell part’, which can be further sub-divided into cascades of ‘intracellular’, ‘cytoplasmic vesicle’ and ‘intrinsic to membrane’. In the next main sub-category of ‘Molecular Function’, ‘ion binding’ and ‘catalytic’ were the most abundant cascades that have a respective of 71 and 19 genes. Moreover, ‘hydrolase activity acting on glycosyl bonds’ and ‘iron ion binding’ were the two dominant groups in the cascade of ‘catalytic’. Within the last sub-category ‘Biological Process’, ‘cellular process’ and ‘metabolic process’ were the two most prevalent cascades that can represent the typical activities of biological processes. Specifically, the most intriguing GO terms in ‘cellular process’ were found to be ‘meiosis I’ and ‘pollen wall assembly’, suggesting their active roles in MS. It was noted that ‘DNA recombination’ was highlighted in the cascade ‘metabolic process’.

In the KEGG analysis, a total of 34 pathways were enriched, of which 13 were inferred from both up- and down- regulated genes, and the rest were inferred from either down- or up- regulated genes alone (Table 2). It was showed that most of the genes are involved in ‘Metabolic pathways’ and ‘Biosynthesis of secondary metabolites’. Interestingly, there were at least 6 genes (SIN_1006103, SIN_1017099, SIN_1014074, SIN_1023392, SIN_1015497 and SIN_1014349) annotated as ‘Meiosis-yeast’ or ‘Oocyte meiosis’ in the list of genes down-regulated in sterile buds, consistent with the GO annotation results. In the ‘Biosynthesis of secondary metabolites’ pathway, the number of up-regulated genes was nearly 3 times that of down-regulated genes. Also, many more up-regulated genes were annotated as ‘Polycyclic aromatic hydrocarbon degradation’ and ‘alpha-Linolenic acid metabolism’. By contrast, many genes down-regulated in sterile buds were enriched in ‘Ascorbate and aldarate metabolism’ and ‘Glycerophospholipid metabolism’. There were also 14 up-regulated genes involved in the pathway of ‘Flavonoid biosynthesis’ (Table 2).

**Table 2:** Summary of KEGG annotations for up- and down-regulated genes

Pathway ID	Pathway terms	Down	Up
ko01100	Metabolic pathways	112	116
ko01110	Biosynthesis of secondary metabolites	38	96
ko00500	Starch and sucrose metabolism	23	22
ko04626	Plant-pathogen interaction	18	38
ko04075	Plant hormone signal transduction	18	21
ko00040	Pentose and glucuronateinterconversions	17	11
ko01120	Microbial metabolism in diverse environments	14	22
ko00940	Phenylpropanoid biosynthesis	11	29
ko00627	Aminobenzoate degradation	8	13
ko00945	Stilbenoid, diarylheptanoid and gingerol biosynthesis	6	23
ko00360	Phenylalanine metabolism	6	9
ko00906	Carotenoid biosynthesis	6	15
ko03010	Ribosome	15	
ko00240	Pyrimidine metabolism	14	
ko00230	Purine metabolism	12	
ko04113	Meiosis−yeast	11	
ko00053	Ascorbate and aldarate metabolism	10	
ko04141	Protein processing in endoplasmic reticulum	8	11
ko04111	Cell cycle−yeast	8	
ko04810	Regulation of actin cytoskeleton	7	
ko03008	Ribosome biogenesis in eukaryotes	7	
ko00564	Glycerophospholipid metabolism	7	
ko04110	Cell cycle	7	
ko04114	Oocyte meiosis	6	
ko00250	Alanine, aspartate and glutamate metabolism	6	
ko00941	Flavonoid biosynthesis		14
ko00363	Bisphenol degradation		13
ko00624	Polycyclic aromatic hydrocarbon degradation		12
ko00903	Limonene and pinene degradation		12
ko00908	Zeatin biosynthesis		10
ko00460	Cyanoamino acid metabolism		8
ko00350	Tyrosine metabolism		7
ko00950	Isoquinoline alkaloid biosynthesis		6
ko00592	alpha-Linolenic acid metabolism		6
subtotal		395	514

Down: down-regulated genes; Up: up-regulated genes

**Fig. 3:**
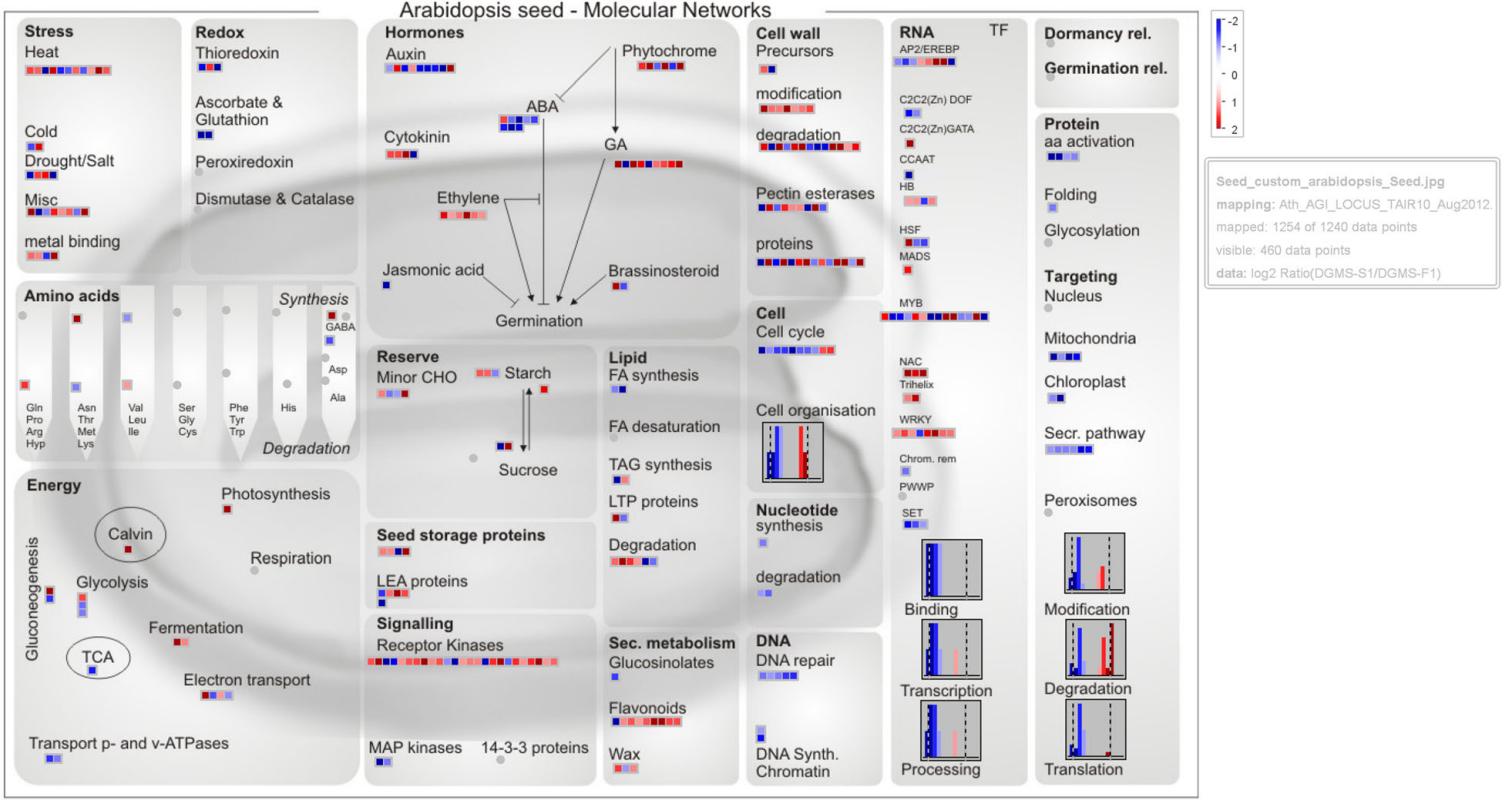
Global view of DEGs involved in diverse metabolic pathways. Differentially expressed genes (DEGs) were selected for the metabolic pathways analysis using the MapMan software (3.6.0RC1). The colored boxes indicate the Log2 ratio of fold changes of DEGs

These findings were further supported by a more specific comparison of metabolic pathways by using MapMan [32]. All of the 1,502 DEGs identified between sterile and fertile buds were annotated in the TAIR database (http://www.arabidopsis.org). Consequently, 1,445 DEGs were found to be homologs of 1,240 Arabidopsis genes (Additional file 2: Table S3). To dissect the putative functions of the 1,445 DEGs that are likely to be associated with MS phenotype, we fully visualized the Arabidopsis homologous genes with MapMan and inferred a candidate pathway network (Fig. 3).

In the network, the most significant changes in transcript abundance of genes were shown to be related to ‘Protein’, ‘Targeting’, ‘Hormones’ and ‘DNA’. Moreover, the expression of genes implicated in ‘Ethylene and JA synthesis’ were up-regulated in sterile buds, while those genes involved in ‘Signaling pathway’ were down-regulated in the DGMS sterile buds. In addition, the DEGs involved in ‘Lipid (FA synthesis)’, ‘Redox (Ascorbate & Glutathion)’ and ‘Energy (transport p- and v-ATPases)’ were all down-regulated, whereas those in ‘Second Metabolism (Flavonoids)’, ‘Cell Wall (Modification)”, and ‘Energy (Fermentation)’ were up-regulated in sterile buds, if compared to those in fertile buds. Among the differentially expressed transcription factors within the ‘RNA TF’ group, all of the NAC, trihelix and WRKYs (except one WRKY) were up-regulated, whereas C2C2(Zn) DOF, CCAAT and SET were down-regulated. Furthermore, in the ‘Signalling’ category, two MAP kinase-coding genes were down-regulated in the sterile buds (Fig. 3; Additional file 2: Table S3).

### Identification of male-sterility/male-reproduction related genes

To gain a deeper insight into the molecular mechanism underlying MS, we queried the sesame DEGs in the PMRD which contains 548 Arabidopsis male-sterility/male-reproduction related genes. Forty nine homologous genes related to plant male reproduction were retrieved; several of these genes encode transcription factors (e.g. bHLH089, MYB99 and AMS). The transcription factor encoding genes showed reduced expressions in sterile buds, implicating their important roles in specifying/determining tapetal fate and development (Table 3).

**Table 3:** Sesame DEGs homologous to Arabidopsis male-sterility/reproduction genes

Query ID^a^	log_2_FC	Subject ID	E-value	Score	Symbol	Description
Down-regulated, homologs of MS gene (cloned)^b^						
SIN_1005144	−10.88	AT4G39910	1E-22	90.1	ATUBP3	ubiquitin-specific protease 3 (UBP3)
SIN_1012023	−8.47	AT1G69940	2E-140	407	AtPPME1	PPME1
SIN_1020647	−4.94	AT4G02780	0	634	ABC33, CPS1, GA1	GA REQUIRING 1 (GA1)
SIN_1015972	−4.78	AT1G65470	2E-79	264	FAS1, NFB2	FASCIATA 1 (FAS1)
SIN_1008904	−2.36	AT1G77850	3E-139	418	ARF17	auxin response factor 17 (ARF17)
SIN_1001388	−2.07	AT4G21330	2E-36	129	DYT1	DYSFUNCTIONAL TAPETUM 1 (DYT1)
SIN_1013503	−1.94	AT3G07970	4E-103	315	QRT2	QUARTET 2 (QRT2)
SIN_1007044	−1.67	AT5G54680	6E-61	194	bHLH105, ILR3	iaa-leucine resistant3 (ILR3)
SIN_1020616	−1.61	AT2G24450	1E-41	146	FLA3	FASCICLIN-like arabinogalactan protein 3 (FLA3)
SIN_1014349	−1.61	AT3G13170	1E-172	488	AtSPO11-1, AtTAF6	ATSPO11-1
SIN_1025211	−1.52	AT5G22130	6E-110	329	PNT1	PEANUT 1 (PNT1)
SIN_1015023	−1.49	AT3G54140	0	768	ATPTR1, PTR1	peptide transporter 1 (PTR1)
SIN_1008202	−1.36	AT4G39400	1.6	26.2	BIN1, BRI1, DWF2	BRASSINOSTEROID INSENSITIVE 1 (BRI1)
SIN_1008272	−1.30	AT3G48750	2E-91	280	CDC2, CDK2	cell division control 2 (CDC2)
SIN_1005880	−1.17	AT3G52590	5E-41	134	ERD16, HAP4, UBQ1	ubiquitin extension protein 1 (UBQ1)
SIN_1013864	−1.14	AT4G23850	0	1022	LACS4	long-chain acyl-CoA synthetase 4 (LACS4)
SIN_1004796	−1.01	AT1G50500	0	875	HIT1, VPS53	HEAT-INTOLERANT 1 (HIT1)
Upregulated, homologs of MS gene (cloned)						
SIN_1004507	1.00	AT4G34850	0	624	LAP5	LESS ADHESIVE POLLEN 5 (LAP5)
SIN_1015330	1.27	AT3G23920	0	852	BAM1, BMY7	beta-amylase 1 (BAM1)
SIN_1003190	1.43	AT1G02205	0	711	CER1	ECERIFERUM 1 (CER1)
SIN_1011100	1.84	AT2G38110	0	735	ATGPAT6, GPAT6	glycerol-3-phosphate acyltransferase 6 (GPAT6)
SIN_1006818	1.97	AT4G20050	0	558	QRT3	QUARTET 3 (QRT3)
SIN_1005329	2.22	AT3G23240	3E-31	114	ATERF1, ERF1	ethylene response factor 1 (ERF1)
SIN_1009935	2.28	AT3G61230	6E-104	303	PLIM2c	GATA type zinc finger transcription factor
SIN_1004029	2.53	AT5G14380	0.015	33.5	AGP6	arabinogalactan protein 6 (AGP6)
Downregulated, homologs of MR gene with mutant evidence						
SIN_1026151	−1.02	AT2G14760	2E-68	221		basic helix-loop-helix (bHLH) DNA-binding protein
SIN_1008063	−1.01	AT1G26610	4E-22	99.8		C2H2-like zinc finger protein
Downregulated, homologs of MR gene with GO evidence						
SIN_1006103	−2.68	AT5G57880	1E-24	101	MPS1, PRD2	MULTIPOLAR SPINDLE 1 (MPS1)
SIN_1006609	−2.46	AT3G47870	1E-37	137	LBD27, SCP	LOB domain-containing protein 27 (LBD27)
SIN_1017022	−1.69	AT1G78130	0	665	UNE2	unfertilized embryo sac 2 (UNE2)
SIN_1014074	−1.63	AT1G01690	2E-48	170	ATPRD3, PRD3	putative recombination initiation defects 3 (PRD3)
SIN_1017944	−1.59	AT1G64110	0	1150		P-loop containing nucleoside triphosphate hydrolases
SIN_1022121	−1.51	AT4G24972	2E-12	61.6	TPD1	TAPETUM DETERMINANT 1 (TPD1)
SIN_1015497	−1.48	AT1G63990	7E-117	345	SPO11-2	sporulation 11–2 (SPO11-2)
SIN_1007667	−1.43	AT4G29170	3E-119	344	ATMND1	ATMND1
SIN_1005858	−1.36	AT5G24330	3E-127	367	ATXR6, SDG34	Arabidopsis Trithorax-Related Protein 6 (ATXR6)
SIN_1015063	−1.22	AT3G44540	1E-153	449	FAR4	fatty acid reductase 4 (FAR4)
SIN_1001377	−1.12	AT1G11330	0	611		S-locus lectin protein kinase family protein
SIN_1009130	−1.06	AT4G25590	8E-89	258	ADF7	actin depolymerizing factor 7 (ADF7)
SIN_1016644	−1.02	AT1G25450	1E-157	461	CER60, KCS5	3-ketoacyl-CoA synthase 5 (KCS5)
Upregulated, homologs of MR gene with GO evidence						
SIN_1014884	1.00	AT4G34050	3E-163	456	CCoAOMT1	caffeoyl coenzyme A O-methyltransferase 1 (CCoAOMT1)
SIN_1002228	1.03	AT3G28470	2E-39	144	TDF1, ATMYB35	Defective In Meristem Development And Function 1 (TDF1)
SIN_1026357	1.30	AT5G23530	4E-101	305	AtCXE18, CXE18	carboxyesterase 18 (CXE18)
SIN_1012979	1.41	AT5G03700	5E-155	454		D-mannose binding lectin protein
SIN_1002445	1.57	AT2G37040	0	1104	ATPAL1, PAL1	PHE ammonia lyase 1 (PAL1)
SIN_1019202	1.66	AT3G59530	0	608	LAP3	Calcium-dependent phosphotriesterase
SIN_1019338	2.29	AT3G17220	3E-08	50.4	ATPMEI2, PMEI2	pectin methylesterase inhibitor 2 (PMEI2)
SIN_1007695	7.68	AT2G19070	0	540	SHT	spermidine hydroxycinnamoyl transferase (SHT)
SIN_1005049	7.69	AT4G27290	0	873		S-locus lectin protein kinase family protein

^a^The homologue search using Blast; ^b^arabidopsis male-sterility/male-reproduction related genes in PMRD (Plant Male Reproduction Database, http://www.pmrd.org/)

### Allelic variants of DEGs

To gain a better understanding of the DEGs, we further predicted the effect of allelic variants on the function of their target genes using SnpEff predictor. A total of 1,057 Insertion/Deletions (InDels) were detected in 982 genes expressed in fertile buds, of which 52 reside within 48 DEGs (some genes have two InDels) (Additional file 3: Table S4). Similarly, 1,432 InDels were detected in 1,354 genes expressed in sterile buds, and 86 InDels were located within 83 DEGs (Additional file 4: Table S5). Together, we identified 138 InDels within 131 genes that were differentially expressed either in fertile or sterile buds. Of the 138 InDels identified, 62 were located in 57 genes that were up-regulated in sterile buds, and 76 were located in 68 genes that were down-regulated in sterile buds (Additional files 5: Table S6 and 6: Table S7).

Specifically, in the list of up-regulated genes, a number of transcription factor encoding genes such as SIN_1002610 (Ethylene-responsive transcription factor ERF106), SIN_1024026 (NAC2), SIN_1019334 (WRKY 28) and SIN_1011023 (WRKY 33) were found. Some genes encoding ‘Brassinosteroid-regulated protein BRU1’ (SIN_1022411), ‘COP9 signalosome complex subunit 2’ (SIN_1015172) and ‘Defensin J1-2’ (SIN_1021298) were also highlighted (Additional file 5: Table S6). In the list of down-regulated genes, SIN_1008339 (E3 ubiquitin-protein ligase MARCH1), SIN_1010740 (L-ascorbate oxidase homolog), SIN_1026145 (Pollen-specific protein SF3), SIN_1005014 (Protein disulfide-isomerase 5–3) and SIN_1010051 (Sugar transport protein 8) were of interested in that they were likely to be related with pollen development (Additional file 6: S7).

A subset of 21 genes containing InDels that were predicted to cause loss of function (LOF) and/or codon change (CC) was selected for further analysis (Table 4). Of these, InDels likely to cause CC (termed ‘CC-type’) were detected in 6 genes at sterile alleles, and in other 6 genes at fertile alleles. Moreover, LOF-type InDels were also detected in 6 fertile alleles and 7 sterile alleles, which showed a higher expression level in fertile buds and sterile buds, respectively (marked with asterisk; Table 4). Thus, it seemed that LOF-Type InDel might lead to the increase of transcript abundance in which it resides. This observation was further confirmed by the fact that in the 11 genes up-regulated in fertile buds, the majority (9 out of 11) of InDels were detected in fertile alleles. Similarly, in the other 10 genes up-regulated in sterile buds, the majority (80 %) of the InDels were detected in sterile alleles.

**Table 4:** 21 DEGs with InDels prone to cause loss of function or codon change

Gene ID	InDel^aLOF^	Codon-change	log_2_(S/F)	Annotation
Allele from sterile buds				
SIN_1025190	c.1151_1151 + 1insCG^a^	p.Gly385fs	10.09	SCP18_ARATH Serine carboxypeptidase-like 18
SIN_1017245	c.435dupA^a^	p.Leu146fs	3.51	F3PH_ARATH Flavonoid 3’-monooxygenase
SIN_1018350	c.889_890insT^a^	p.Thr297fs	1.39	IPT_HUMLU Adenylate isopentenyltransferase
SIN_1013325	c.507_507 + 1insTTTGAAGAT^a^		1.28	AB22G_ARATH ABC transporter G family
SIN_1022131	c.465 + 1_465 + 2insA^a^		1.54	DHI1L_XENTR Hydroxysteroid 11-beta-dehydrogenase 1
SIN_1007649	c.886-2_886-1insT^a^		1.47	BGAL_MALDO Beta-galactosidase
SIN_1001108	c.934-2_934-1insCC^a^		4.33	E13B_WHEAT Glucan endo-1,3-beta-glucosidase
SIN_1010915	c.855dupT	p.Pro286fs	4.20	R1B14_SOLDE late blight resistance protein
SIN_1025700	c.318_319insCTGGAA	p.V106_A107insLE	−1.00	SCL32_ARATH Scarecrow-like protein 32
SIN_1005818	c.597_599delAAG	p.Arg200del	−1.49	HMGB9_ARATH High mobility group B protein 9
Allele from fertile buds				
SIN_1005818	c.394 + 2delT^a^		−1.49	HMGB9_ARATH High mobility group B protein 9
SIN_1004626	c.1008delG^a^	p.Gln336fs	−1.63	Y9955_DICDI serine/threonine-protein kinase
SIN_1009714	c.1245 + 1_1245 + 9delGTTGGTTTC^a^		−1.11	BRCA1_ARATH (BREAST CANCER SUSCEPTIBILITY)
SIN_1008339	c.698 + 1delG^a^		−1.21	MARH1_MOUSE E3 ubiquitin-protein ligase
SIN_1026641	c.750-2delA^a^		−1.25	PGLR2_PLAAC Exopolygalacturonase (Fragment)
SIN_1014879	c.1048-2_1048-1insA^a^		−2.01	Y5713_ARATH PI-PLC X domain-containing protein
SIN_1021763	c.1005_1006delCA	p.His335fs	−1.56	FIP1X_SCHPO Pre-mRNA polyadenylation factor fip1
SIN_1022317	c.712_713dupGT	p.Trp240fs	−1.59	PP323_ARATH Pentatricopeptide repeat protein
SIN_1009152	c.1653_1654insC	p.Ser552fs	−1.08	Y5241_ARATH Probable receptor-like protein kinase
SIN_1004703	c.74delC	p.Ala25fs	1.30	AMERL_ARATH AMMECR1 domain protein
SIN_1019529	c.18_20dupAGG	p.Gly7dup	1.54	DUF3774 Wound-induced protein

^a^ means InDel may cause Loss of function (LOF), as predicted by SnpEff. See Additional file 7: Table S8 for full information

In particular, some genes such as SIN_1025190 (*SCP18*, Serine carboxypeptidase), SIN_1017245 (*F3PH*, Flavonoid 3'-monooxygenase) and SIN_1018350 (*IPT*, Adenylate isopentenyltransferase) with both LOF-type and CC-type InDels in sterile alleles, were up-regulated in sterile buds. Moreover, the gene encoding a kinase (SIN_1004626) with both LOF- and CC- types of InDels in fertile allele was up- regulated in fertile buds (down-regulated in sterile buds). Interestingly, in another gene, SIN_1005818 (HMGB9, High mobility group B protein 9), InDel was detected in both alleles, with putative disruptive_inframe_deletion in sterile allele and LOF in fertile allele. The expression of this gene was down-regulated in sterile buds but up-regulated in fertile buds (Table 4, Additional file 7: Table S8). Taken together, a large number of sequence variants were detected in these DEGs, and their effects on transcript abundances were not conclusive.

### Real-time quantitative PCR validation

To verify the RNA-Seq results, we chose an alternative strategy for both the up- and down-regulated DEGs. Twenty genes were randomly selected for validation by Real-time quantitative PCR (qRT-PCR) using the same RNA samples that was used for RNA-Seq. Primer sets were designed to span exon–exon junctions (Additional file 8: Table S1). Results showed that although genes expression fold changes detected by qRT-PCR, in most cases, were higher than those by RNA-Seq, the trends were similar between these two methods, thus confirming the accuracy and reliability of RNA-Seq. As an example, the expression patterns of 12 randomly selected Male-sterility/male-reproduction genes were listed in Table 5, which demonstrated that the expression levels revealed by qRT-PCR and RNA-Seq were highly correlated (*r* = 0.762, *P* < 0.01, n = 12).

**Table 5:** qRT-PCR verification of sesame male-sterility / reproduction related 12 DEGs detected by RNA-seq

Query ID^a^	Subject ID^a^	E-value^a^	Score^a^	Description	Log_2_FC^b^	S/F^c^
SIN_1007162	AT5G20710	0	1122	BGAL7; beta-galactosidase	2.31	5.87 ± 0.96
SIN_1021282	AT1G14750	4E-107	337	SOLO DANCER, cyclin	−2.67	0.52 ± 0.19
SIN_1016793	AT1G06260	2E-125	367	cysteine proteinase	4.47	20.16 ± 1.82
SIN_1004507	AT4G34850	0	624	chalcone and stilbene synthase	1.00	4.24 ± 2.36
SIN_1001388	AT4G21330	2E-36	129	DYT1, bHLH transcription factor	−2.07	0.48 ± 0.15
SIN_1003502	AT2G42940	1E-90	272	AT-hook DNA-binding protein	3.82	7.00 ± 1.23
SIN_1007695	AT2G19070	0	540	SHT	7.68	13.91 ± 1.03
SIN_1013713	AT3G27730	0	1274	ROCK-N-ROLLERS/AtMER3	−1.57	0.74 ± 0.15
SIN_1020712	AT4G29250	2E-141	416	acyl-transferase family protein	4.40	3.45 ± 4.02
SIN_1022113	AT3G57620	0	670	glyoxal oxidase-related protein	4.04	13.42 ± 0.58
SIN_1006818	AT4G20050	0	558	QRT3; polygalacturonase	1.97	4.34 ± 0.91
SIN_1019202	AT3G59530	0	608	strictosidine synthase	1.66	3.31 ± 0.57

^a^The homologue search using Blast; ^b^RNA-seq Log_2_FC(S/F); ^c^S/F means fold change of gene between sterile bud and fertile bud by qRT-PCR

## Discussion

We presented here, to our knowledge, the first study of sesame DGMS at transcriptome level. Transcript abundances from both fertile and sterile buds were acquired by RNA-Seq using the Illumina sequencing platform. We then mapped the high quality transcriptome reads onto the sesame reference genome and identified more than 22 thousands expressed genes, of which only 1,502 genes (~6.6 %) were differently expressed in either sterile or fertile buds, suggesting that a limited number of key genes are enough to transform the trait observably, although the development of anther is a complicated and polygenic process.

We identified 49 anther development related genes in sesame that have homologs in Arabidopsis, some of which encoded transcription factors (bHLH089, MYB99, and AMS) and were possibly associated with the determination of tapetal fate and development (Table 3). Of these, 32 were down-regulated and the rest of 17 were up-regulated. Moreover, homologs of MS genes (cloned) accounted for nearly one half of the genes within each regulated category, and the rest of genes were annotated as MR related (male-reproduction related genes, with GO evidence), thus demonstrating that all these genes might be good candidates responsible for MS (Table 3). This can be explained by the fact that the sesame MS mentioned here initiated from PMC, the second stage of the anther and pollen development pathway [13], thus leading to the failure of anthers development, as observed in the male sterile buds [8].

Specifically, we found that *DYT1* and *TPD1* were in the list of 32 down-regulated DEGs (Table 3). Previous study has showed that *DYT1* might regulate anther development via the expression of *AMS* and many tapetum-preferential genes, thereby indirectly affects pollen wall formation [17]. TPD1, a small peptide, was mainly expressed in microsporocytes and likely secreted into the interface between the tapetum and male reproductive cells to interact and form a receptor complex with the leucine-rich repeat receptor-like kinases EMS1, thus determining cell fate of the tapetal layer [16, 33]. Therefore, it is likely that the down regulation of *DYT1* and *TPD1* in sesame might affect the pollen release through determining cell fate of the tapetal layer.

Another gene of interest was *RBOHE* (RESPIRATORY BURST OXIDASE HOMOLOGUE E). Previous study also showed that *RBOHE* (At1g19230) was an anther-preferential or tapetum-enriched gene, and functional loss of *RBOHE* resulted in delayed tapetal degeneration, thus the expression of *RBOHE* was reduced in *dyt1* and *tdf1* [33]. Consistent with this, we found that the *RBOHE* homologs in sesame, SIN_1024646 and SIN_1007549, also displayed significantly reduced expression in sterile buds (log_2_S/F = −1.7 and −0.9), if compared to fertile buds (Additional file 1: Table S2). Therefore, *RBOHE* may have a similar function in sesame DGMS.

Apart from *DYT1* mentioned above, *QRT2* (QUARTET2) was also in the MS genes (cloned) list (Table 3). Three *QRT* genes including *QRT2* are required for the degradation of pollen mother cell wall when microspores are released from their tetrads [12]. Furthermore, *QRT2* are required for anther dehiscence. In the process of floral abscission which co-regulated by JA, ethylene and abscisic acid (ABA), *QRT2* is regulated by ethylene and ABA [34]. Moreover, anther dehiscence-related polygalacturonase activity is likely to be regulated by JA, ethylene and ABA [13]. In this study, the reduced expression of *QRT2* was coupled with the up-regulation of genes involved in ethylene synthesis.

There were 17 up-regulated sesame genes with homologs in Arabidopsis (8 homologous to MS genes and 9 to MR genes, Table 3). Of these, the expression level of SIN_1007695 (*spermidine hydroxycinnamoyl transferase*, *SHT*) showed >200 fold increase in sterile buds, which was reminiscent of *SHT* expressed in the tapetum of Arabidopsis anthers [35]. Moreover, *SHT* was assigned into ‘cluster 81’ by the online tool of FlowerNet [36], which includes several genes such as KCS10, GH31 and ATA7; their homologs in sesame (i.e. SIN_1007525, SIN_1025709 and SIN_1002500) were co-up-regulated in sterile buds (Additional file 1: Table S2), implying their possible involvement in MS. This ‘cluster 81’ also contained *TSM1* (*tapetum-specific methyltransferase1*), which encodes a cation-dependent CCoAOMT-like protein involved in phenylpropanoid polyamine conjugate biosynthesis and has a role in the stamen/pollen development of Arabidopsis [37]; the rest of genes with unknown functions are likely to play roles in pollen exine and lipid biosynthesis, based on their description in AtEnsembl [36]. Therefore, it would be worthy of investigating the rest genes within this cluster to get a clear view of their function.

JA is specifically required for anther dehiscence during anther development [38]. Mutations in genes that participate in JA biosynthesis and perception cause a failure or delay in anther dehiscence and pollen inviability which result in male sterility [39]. Examples of such genes include the *DEFECTIVE IN ANTHER DEHISCENCE 1* (*DAD1*), which encodes a phospholipase A1 that catalyses the initial step of JA biosynthesis; *AOS*, a gene that encodes allene oxide synthase; *DEHISCENCE 1* (*DDE1*)/*OPR3*, which encodes the OPR protein 12-oxo-phytodienoic acid reductase in the JA synthesis pathway [40]. Defects in all stages of the JA pathway appear to cause similar phenotypes of reduced filament elongation and a lack of dehiscence. Delayed dehiscence or non-dehiscence phenotypes have been observed in mutants defective in JA biosynthetic enzymes [13]. In this study, SIN_1016850 (homolog of *PLA15*, *Phospholipase A1-Igamma1*) was significantly up-regulated in sterile buds, whereas the homologs of allene oxide synthase encoding genes did not show differences (data not shown). However, SIN_1022877 and SIN_1022878, which are homologs of *OPR1* (*12-oxophytodienoate reductase 1*) in Arabidopsis, displayed obvious down-regulation in sterile buds (Additional file 1: Table S2). These data strongly indicated that genes involved in JA pathway are also responsible for MS in sesame.

Plant gene expression regulation is a complicated network. Through specific interactions with cis-acting target elements, transcription factors can regulate a series of relevant down-stream targets, which play an important role in plant development and the response to environmental stress. Arabidopsis *ANTHER INDEHISCENCE FACTOR* (*AIF*), a NAC-like gene, acts as a repressor that controls anther dehiscence by regulating genes in the jasmonate biosynthesis [38]. In fact, for the annotated NACs in Swissprot, all of the 9 sesame homologs were up-regulated in sterile buds, which strengthen the role of NACs in the regulation of MS (Fig. 3, Additional file 1: Table S2). Furthermore, 11 of the 12 WRKYs that were significantly up-regulated in sterile buds, were annotated as the orthologs of WRKY33 (Fig. 3, Additional files 1: Table S2). WRKY33 proteins are evolutionarily conserved with a critical role in broad plant stress responses, and Arabidopsis WRKY33 is a key transcriptional regulator of hormonal and metabolic responses [41]. Moreover, genes involved in redox homeostasis, salicylic acid (SA) signaling, ethylene-JA-mediated cross-communication and camalexin biosynthesis were identified as direct targets of WRKY33 [42]. Furthermore, the down-regulation of JA-associated responses appears to involve direct activation of several jasmonate ZIM-domain genes, encoding repressors of the JA-response pathway, by loss of WRKY33 function and by additional SA-dependent WRKY factors. In the present study, the co-expression behavior of NACs and WRKYs suggested their pivotal roles in regulating the sesame MS (Fig. 3, Additional file 1: Table S2).

To understand the impact of sequence variation on gene expression, the effects of allelic variants on the function of their target genes were predicted using SnpEff. Interestingly, 6 InDels were found in fertile alleles, which were up-regulated in fertile buds (and the wild-type sterile allele had lower level of expression in sterile buds); and 7 InDels were found in sterile alleles, which were up-regulated in sterile buds (Table 4). This observation suggested that the causal effect of sequence variation on transcript abundance was not so straightforward, but rather confound. This can be explained by the way that most of the InDels were detected in coding regions rather than in the promoter regions, in which it can directly affect the transcript abundance. Occasionally, we also identified InDels showing a transcriptional-regulatory function, in which the transcript abundance was decreased by the existing of causative InDels. For example, two genes (SIN_1025700 and SIN_1005818) with InDels in sterile alleles caused a decrease of transcript abundances in sterile buds, and another two genes (SIN_1004703 and SIN_1019529) with InDels in fertile alleles led to the down-regulation of genes in fertile buds, thus demonstrating a cis-acting fashion.

As suggested by Rutley and Twell [43], transcriptome studies of the male gametophyte have not only increased our knowledge and understanding, but also improved the efficacy of experimental strategies by informing experimental design (such as by gene selection for reverse genetics) and through query-based and co-expression analysis. The present investigation provided many DEGs and a number of candidate genes that can be used to elucidate the molecular mechanism underlying sesame DGMS through transgenic verification in future.

## Conclusions

This study provided a set of 1,502 genes differentially expressed in the fertile and sterile buds of sesame DGMS lines based on transcriptome profiling. Half of these genes were up-regulated in sterile buds, demonstrating a complex expression pattern. Regarding the genes implicated in ethylene and JA synthesis & signaling, the expression of which were up- and down- regulated in the sterile buds, respectively. Furthermore, the majority of NAC and WRKY transcription factors were up-regulated in sterile buds.

Moreover, 49 sesame genes with homologs in Arabidopsis related with male-sterility/male-reproduction showed reduced expression in sterile flower. Some of these genes encode transcription factors (bHLH089, MYB99, and AMS) that possibly have a role in specifying or determining tapetal fate and development. Furthermore, the predicted effect of allelic variants on the function of target gene highlighted several InDels, which might contribute to fertility determination.

## Methods

### Plant materials and RNA preparation

The sesame plant materials used in this study include the newly developed DGMS line W1098A and its fertile counterpart W1098B, which differed from each other only by pollen fertility [8]. These two lines were both cultivated in the experimental fields of the Oil Crops Research Institute, CAAS (Wuhan, Hubei Province, China). Buds with a length of ~2.5 mm were separately stripped from each of five male sterile and fertile plants and bulked for transcriptomic profiling. The fertile bulk and the sterile bulk of buds were immediately snap-frozen in liquid nitrogen and then stored at −80 °C freezer until use. Total RNA was isolated from bulks of sterile buds and fertile buds with TRIzol reagent (Gibco-BRL) according to the manufacturer’s instruction. Then two cDNA libraries were constructed from sterile and fertile buds, as previously described in sesame [44]. Briefly, approximately 5 mg of mRNA was fragmented, converted to cDNA, and PCR amplified according to the Illumina RNA-Seq protocol (Illumina, Inc. San Diego, CA). Sequence reads were generated using the Illumina Genome AnalyzerII (SanDiego, CA) and Illumina HiSeq 2000 platform (San Diego, CA) at the Beijing Genomics Institute (Shen Zhen, China).

### Identification of Differentially Expressed Genes

The clean reads were mapped to the reference genome sequence of *S. indicum* (http://ocri-genomics.org/Sinbase/) [11] using SOAP aligner/soap2 (an improved ultrafast tool for short read alignment) [45]. RPKM were used to gauge the relative transcript abundance for each gene. Using the DEGseq program, significantly differential gene expression was identified between the fertile and sterile buds libraries [46]. The FDR was used to determine the threshold *p*-value. In this study, a stringent of FDR ≤ 0.001 and │log2 (Fold change ratio of sterile/fertile)│ ≥ 1.00 was used as the threshold to select a significantly different expressed gene.

### Characterization of genetic variations

Characterization of the sequence variants such as InDels was performed using SnpEff version 4.1 [47] by referring to sesame genome annotation downloaded from the Sinbase (http://ocri-genomics.org/Sinbase) according to Wang et al. [48]. Sequence variants (InDels, frame shift, stop gained, stop lost and non synormymous coding) that potentially have high impact on transcript/protein were predicted according to the method described by Saeed et al. [49].

### GO and KEGG Pathway Enrichment Analysis

The DEGs were used for GO and pathway enrichment analysis. A corrected *P* ≤ 0.05 was selected as the threshold of significance to determine enrichment in the gene sets [50]. Functional classes inferred from DEGs were assigned according to GO mapping provided by the ensemble database. The Blast2GO program (https://www.blast2go.com/) was used to obtain GO annotations for the all DEGs [51]. Then, the results were submitted to WEGO (http://wego.genomics.org.cn) to generate a GO classification graph of all DEGs [52].

KEGG pathway analysis was based on the comparative results between our maped genes and the current KEGG database [53]. MapMan (version 3.5.1 R2) was also used to annotate the DEGs onto metabolic pathways.

### Confirmation of candidate DEGs by qRT-PCR

To validate the DEGs detected by RNA-seq, 20 DEGs were randomly selected from 52 common differentially expressed genes in two libraries and then subjected to qRT-PCR analysis, according to Qi et al. [54]. Gene-specific primers were designed with the online tool Primer3 [55] based on the selected unigenes sequences (Additional file 8: Table S1). Reactions were performed with the SYBR Green Real time PCR Master Mix (TOYOBO, Japan) in a Bio-Rad CFX96 instrument. For each sample, three replicates were run for each gene in a 96-well plate. The relative expression level of each gene was determined using the 2^−ΔΔC^_T_ method [56]. All data are expressed as mean ± standard deviation.

## Additional files

Additional file 1: Table S2. Expressions and annotations of the 1502 differentially expressed unigenes in sesame. (XLSX 338 kb)
Additional file 2: Table S3. Blast results of sesame 1445 DEGs in MapMan pathway analysis. (XLSX 264 kb)
Additional file 3: Table S4. 52 DEGs with InDels detected in fertile buds. (XLSX 28 kb)
Additional file 4: Table S5. 86 DEGs with InDels detected in sterile buds. (XLSX 37 kb)
Additional file 5: Table S6. 62 InDels in 57 DEGs up-regulated in sterile buds. (XLSX 29 kb)
Additional file 6: Table S7. 77 InDels in 68 DEGs down-regulated in sterile buds. (XLSX 33 kb)
Additional file 7: Table S8. The predicted effects of InDels in DEGs. The sequence variations (i.e. InDels) were first detected between sterile and fertile alleles and their potential effects (i.e. causing loss of gene function or codon change) were then predicted by the software SnpEff. (XLSX 15 kb)
Additional file 8: Table S1. List of primer sequences for qRT-PCR. (XLSX 11 kb)

## Abbreviations

ABA: Ethylene and abscisic acid
*AIF*: *ANTHER INDEHISCENCE FACTOR*
AMS: ABORTED MICROSPORES (AMS)
*AOS*: Allene oxide synthase
CMS: Cytoplasmic male sterility
*DAD1*: *DEFECTIVE IN ANTHER DEHISCENCE 1*
*DDE1*: *DEHISCENCE 1* (also known as *OPR3*)
DEGs: Differentially expressed genes
DGMS: Dominant genic male sterile line
DYT1: DYSFUNCTIONAL TAPETUM1
EMS1: EXCESS MICROSPOROCYTES1 (also known as EXTRA SPOROGENOUS CELLS)
FDR: False discovery rate
FDR: False discovery rate
GMS: Genic male sterility
GO: Gene Ontology
GO: Gene O*ntology*
HMGB9: High mobility group B protein 9
InDel: Insertion/Deletion
IPT: Adenylate isopentenyltransferase
JA: Jasmonic acid
*KEGG*: *Kyoto enc*yclopedia of genes and genomes
LG: Linkage group
LG: Linkage groups
LOF: Loss of function
MS: Male sterility
MS: Male sterility
*MS1*: *MALE STERILITY1*
*OPR1*: *12-oxophytodienoate reductase 1*
PCD: Programmed cell death
*PLA15*: *Phospholipase A1-Igamma1*
PMC: Pollen mother cell
PMRD: Plant Male Reproduction Database
QRT: QUARTET
QRT2: QUARTET2
qRT-PCR: Real-time quantitative PCR
RBOH: Respiratory burst oxidase homologue
*RBOHE*: RESPIRATORY BURST OXIDASE HOMOLOGUE E
RPKM: Reads per Kilobase of exon model per Million mapped reads
RPKM: Reads per Kilobase of exon model per Million mapped reads
SA: Salicylic acid
*SHT*: *Spermidine hydroxycinnamoyl transferase*
SHT: Spermidine hydroxycinnamoyl transferase
*TDF1*: *DEFECTIVE IN TAPETAL DEVELOPMENT AND FUNCTION1*
TDF1: TAPETAL DEVELOPMENT AND FUNCTION1
TPD1: TAPETAL DETERMINANT1

## References

[1] Karatzi K, Stamatelopoulos K, Lykka M, Mantzouratou P, Skalidi S, Zakopoulos N (2013). Sesame oil consumption exerts a beneficial effect on endothelial function in hypertensive men. Eur J Prev Cardiol.

[2] Periasamy S, Hsu DZ, Chang PC, Liu MY (2014). Sesame oil attenuates nutritional fibrosing steatohepatitis by modulating matrix metalloproteinases-2, 9 and PPAR-gamma. J Nutr Biochem.

[3] Anilakumar KR, Pal A, Khanum F, Bawa AS (2010). Nutritional, medicinal and industrial uses of sesame (*Sesamum indicum* L.) seeds-an overview. Agric Conspec Sci (ACS).

[4] Uzun B, Arslan C, Furat S (2008). Variation in fatty acid compositions, oil content and oil yield in a germplasm collection of sesame (*Sesamum indicum* L.). J Am Oil Chem Soc.

[5] Erbas M, Sekerci H, Gul S, Furat S, Yol E, Uzon B (2009). Changes in total antioxidant capacity of sesame (*Sesamum indicum* L.) by variety. Asian J Chem.

[6] Murty DS (1975). Heterosis, combining ability and reciprocal effects for agronomic and chemical characters in Sesamum. Theor Appl Genet.

[7] Zheng YZ, Zhang HY, Mei HX, Zhang TD, Wei SL (2003). Advances in Chinese hybrid sesame research. J Henan Agric Sci.

[8] Liu HY, Zhou XA, Wu K, Yang MM, Zhao YZ (2015). Inheritance and molecular mapping of a novel dominant genic male-sterile gene in *Sesamum indicum* L. Mol Breed.

[9] Zhang H, Miao H, Wang L, Qu L, Liu H, Wang Q, Yue M (2013). Genome sequencing of the important oilseed crop *Sesamum indicum* L. Genome Biol.

[10] Wei X, Liu K, Zhang Y, Feng Q, Wang L, Zhao Y, Li D, Zhao Q, Zhu X, Zhu X, Li W, Fan D, Gao Y, Lu Y, Zhang X, Tang X, Zhou C, Zhu C, Liu L, Zhong R, Tian Q, Wen Z, Weng Q, Han B, Huang X, Zhang X (2015). Genetic discovery for oil production and quality in sesame. Nat Commun.

[11] Wang L, Yu S, Tong C, Zhao Y, Liu Y, Song C, Zhang Y, Zhang X, Wang Y, Hua W, Li D, Li D, Li F, Yu J, Xu C, Han X, Huang S, Tai S, Wang J, Xu X, Li Y, Liu S, Varshney RK, Wang J, Zhang X (2014). Genome sequencing of the high oil crop sesame provides insight into oil biosynthesis. Genome Biol.

[12] Cui X, Wang Q, Yin W, Xu H, Wilson ZA, Wei C, Pan S, Zhang D (2012). PMRD: a curated database for genes and mutants involved in plant male reproduction. BMC Plant Biol.

[13] Wilson ZA, Song J, Taylor B, Yang C (2011). The final split: the regulation of anther dehiscence. J Exp Bot.

[14] Kawanabe T, Ariizumi T, Kawai-Yamada M, Uchimiya H, Toriyama K (2006). Abolition of the tapetum suicide program ruins microsporogenesis. Plant and Cell Physiol.

[15] Parish RW, Li SF (2010). Death of a tapetum: a programme of developmental altruism. Plant Sci.

[16] Jia G, Liu X, Owen HA, Zhao D (2008). Signaling of cell fate determination by the TPD1 small protein and EMS1 receptor kinase. Proc Natl Acad Sci U S A.

[17] Zhang W, Sun YL, Timofejeva L, Chen C, Grossniklaus U, Ma H (2006). Regulation of Arabidopsis tapetum development and function by DYSFUNCTIONAL TAPETUM (DYT1) encoding a putative bHLH transcription factor. Development.

[18] Zhu J, Chen H, Li H, Gao JF, Jiang H, Wang C, Guan YF, Yang ZN (2008). Defective in Tapetal development and function 1 is essential for anther development and tapetal function for microspore maturation in Arabidopsis. Plant J.

[19] Xu J, Yang C, Yuan Z, Zhang D, Gondwe MY, Ding Z, Liang W, Zhang D-B, Wilson ZA (2010). The ABORTED MICROSPORES regulatory network is required for postmeiotic male reproductive development in *Arabidopsis thaliana*. Plant Cell.

[20] Yang C, Vizcay-Barrena G, Conner K, Wilson ZA (2007). MALE STERILITY1 is required for tapetal development and pollen wall biosynthesis. Plant Cell.

[21] Chen C, Farmer AD, Langley RJ, Mudge J, Crow JA, May GD, Huntley J, Smith AG, Retzel EF (2010). Meiosis-specific gene discovery in plants: RNA-Seq applied to isolated Arabidopsis male meiocytes. BMC Plant Biol.

[22] Logacheva MD, Kasianov AS, Vinogradov DV, Samigullin TH, Gelfand MS, Makeev VJ, Penin AA (2011). De novo sequencing and characterization of floral transcriptome in two species of buckwheat (*Fagopyrum*). BMC Genomics.

[23] Wei M, Song M, Fan S, Yu S (2013). Transcriptomic analysis of differentially expressed genes during anther development in genetic male sterile and wild type cotton by digital gene-expression profiling. BMC Genomics.

[24] Wu Y, Min L, Wu Z, Yang L, Zhu L, Yang X, Yuan D, Guo X, Zhang X (2015). Defective pollen wall contributes to male sterility in the male sterile line 1355A of cotton. Sci Rep.

[25] Fang W, Zhao F, Sun Y, Xie D, Sun L, Xu Z, Zhu W, Yang L, Zhao Y, Lv S, Tang Z, Nie L, Li W, Hou J, Duan Z, Yu Y, Yang X (2015). Transcriptomic Profiling Reveals Complex Molecular Regulation in Cotton Genic Male Sterile Mutant Yu98-8A. PLoS One.

[26] Rhee SJ, Seo M, Jang YJ, Cho S, Lee GP (2015). Transcriptome profiling of differentially expressed genes in floral buds and flowers of male sterile and fertile lines in watermelon. BMC Genomics.

[27] Li J, Han S, Ding X, He T, Dai J, Yang S, Gai J (2015). Comparative transcriptome analysis between the cytoplasmic male sterile line NJCMS1A and its maintainer NJCMS1B in Soybean (*Glycine max* (L.) Merr.). PLoS One.

[28] Yan X, Dong C, Yu J, Liu W, Jiang C, Liu J, Hu Q, Fang X, Wei W (2013). Transcriptome profile analysis of young floral buds of fertile and sterile plants from the self-pollinated offspring of the hybrid between novel restorer line NR1 and Nsa CMS line in *Brassica napus*. BMC Genomics.

[29] An H, Yang Z, Yi B, Wen J, Shen J, Tu J, Ma C, Fu T (2014). Comparative transcript profiling of the fertile and sterile flower buds of pol CMS in *B*. Napus BMC Genomics.

[30] Qu C, Fu F, Liu M, Zhao H, Liu C, Li J, Tang Z, Xu X, Qiu X, Wang R, Lu K (2015). Comparative transcriptome analysis of recessive male sterility (RGMS) in sterile and fertile *Brassica napus* lines. PLoS One.

[31] Ma Y, Kang J, Wu J, Zhu Y, Wang X (2015). Identification of tapetum-specific genes by comparing global gene expression of four different male sterile lines in *Brassica oleracea*. Plant Mol Biol.

[32] Usadel B, Poree F, Nagel A, Lohse M, Czedik-Eysenberg A, Stitt M (2009). A guide to using MapMan to visualize and compare Omics data in plants: a case study in the crop species, Maize. Plant Cell Environ.

[33] Zhao X, de Palma J, Oane R, Gamuyao R, Luo M, Chaudhury A, Hervé P, Xue Q, Bennett J (2008). OsTDL1A binds to the LRR domain of rice receptor kinase MSP1, and is required to limit sporocyte numbers. Plant J.

[34] Ogawa M, Kay P, Wilson S, Swain SM (2009). ARABIDOPSIS DEHISCENCE ZONE POLYGALACTURONASE1 (ADPG1), ADPG2, and QUARTET2 are Polygalacturonases required for cell separation during reproductive development in Arabidopsis. Plant Cell.

[35] Grienenberger E, Besseau S, Geoffroy P, Debayle D, Heintz D, Lapierre C, Pollet B, Heitz T, Legrand M (2009). A BAHD acyltransferase is expressed in the tapetum of Arabidopsis anthers and is involved in the synthesis of hydroxycinnamoyl spermidines. Plant J.

[36] Pearce S, Ferguson A, King J, Wilson ZA (2015). FlowerNet: a gene expression correlation network for anther and pollen development. Plant Physiol.

[37] Fellenberg C, Milkowski C, Hause B, Lange PR, Böttcher C, Schmidt J, Vogt T (2008). Tapetum-specific location of a cation-dependent O-methyltransferase in *Arabidopsis thaliana*. Plant J.

[38] Shih CF, Hsu WH, Peng YJ, Yang CH (2014). The NAC-like gene ANTHER INDEHISCENCE FACTOR acts as a repressor that controls anther dehiscence by regulating genes in the jasmonate biosynthesis pathway in Arabidopsis. J Exp Bot.

[39] Jewell JB, Browse J (2016). Epidermal jasmonate perception is sufficient for all aspects of jasmonate-mediated male fertility in Arabidopsis. Plant J.

[40] Peng YJ, Shih CF, Yang JY, Tan CM, Hsu WH, Huang YP, Liao PC, Yang CH (2013). A RING-type E3 ligase controls anther dehiscence by activating the jasmonate biosynthetic pathway gene DEFECTIVE IN ANTHER DEHISCENCE1 in Arabidopsis. Plant J.

[41] Birkenbihl RP, Diezel C, Somssich IE (2012). Arabidopsis WRKY33 is a key transcriptional regulator of hormonal and metabolic responses toward *Botrytis cinerea* infection. Plant Physiol.

[42] Zhou J, Wang J, Zheng Z, Fan B, Yu JQ, Chen Z (2015). Characterization of the promoter and extended C-terminal domain of Arabidopsis WRKY33 and functional analysis of tomato WRKY33 homologues in plant stress responses. J Exp Bot.

[43] Rutley N, Twell D (2015). A decade of pollen transcriptomics. Plant Reprod.

[44] Wei W, Qi X, Wang L, Zhang Y, Hua W, Li D, Lv H, Zhang X (2011). Characterization of the sesame (*Sesamum indicum* L.) global transcriptome using Illumina paired-end sequencing and development of EST-SSR markers. BMC Genomics.

[45] Li R, Yu C, Li Y, Lam TW, Yiu SM, Kristiansen K, Wang J (2009). SOAP2: an improved ultrafast tool for short read alignment. Bioinformatics.

[46] Wang L, Feng Z, Wang X, Wang X, Zhang X (2010). DEGseq: an R package for identifying differentially expressed genes from RNAseq data. Bioinformatics.

[47] Cingolani P, Platts A, le Wang L, Coon M, Nguyen T, Wang L, Land SJ, Lu X, Ruden DM (2012). A program for annotating and predicting the effects of single nucleotide polymorphisms, SnpEff: SNPs in the genome of *Drosophila melanogaster* strain w1118; iso-2; iso-3. Fly (Austin).

[48] Wang L, Yu J, Li D, Zhang X (2015). Sinbase: an integrated database to study genomics, genetics and comparative genomics in *Sesamum indicum*. Plant Cell Physiol.

[49] Saeed B, Baranwal VK, Khurana P (2016). Comparative transcriptomics and comprehensive marker resource development in mulberry. BMC Genomics.

[50] Gao Y, Xu H, Shen Y, Wang J (2013). Transcriptomic analysis of rice (*Oryza sativa*) endosperm using the RNA-Seq technique. Plant Mol Biol.

[51] Conesa A, Götz S (2008). Blast2GO: A comprehensive suite for functional analysis in plant genomics. Int J Plant Genomics.

[52] Ye J, Fang L, Zheng H, Zhang Y, Chen J, Zhang Z, Wang J, Li S, Li R, Bolund L, Wang J (2006). WEGO: a web tool for plotting GO annotations. Nucleic Acids Res.

[53] Kanehisa M, Sato Y, Kawashima M, Furumichi M, Tanabe M (2016). KEGG as a reference resource for gene and protein annotation. Nucleic Acids Res.

[54] Qi X, Xie S, Liu Y, Yi F, Yu J (2013). Genome-wide annotation of genes and noncoding RNAs of foxtail millet in response to simulated drought stress by deep sequencing. Plant Mol Biol.

[55] Untergasser A, Cutcutache I, Koressaar T, Ye J, Faircloth BC, Remm M, Rozen SG (2012). Primer3--new capabilities and interfaces. Nucleic Acids Res.

[56] Livak KJ, Schmittgen TD (2001). Analysis of relative gene expression data using real-time quantitative PCR and the 2^−ΔΔC^_T_ method. Methods.

